# Synthesis, molecular docking evaluation for LOX and COX-2 inhibition and determination of in-vivo analgesic potentials of aurone derivatives

**DOI:** 10.1016/j.heliyon.2024.e29658

**Published:** 2024-04-20

**Authors:** Muhammad Ikram, Ismail Shah, Haya Hussain, Ehsan Ullah Mughal, Nafeesa Naeem, Amina Sadiq, Yasir Nazir, Syed Wadood Ali Shah, Muhammad Zahoor, Riaz Ullah, Essam A. Ali, Muhammad Naveed Umar

**Affiliations:** a Department of Pharmacy, Abdul Wali Khan University Mardan (AWKUM), Mardan, 23390, Pakistan; b Department of Pharmacy, Shaheed Benazir Bhutto University Sheringal, Dir (Upper) 18000, Khyber Pakhtunkhwa, Pakistan; c Department of Chemistry, University of Gujrat, Gujrat, 50700, Pakistan; d Department of Chemistry, Govt. College Women University, Sialkot, 51300, Pakistan; e Department of Chemistry, University of Sialkot, Sialkot, 51300, Pakistan; f Department of Pharmacy, University of Malakand, Chakdara, Chakdara 18800, Khyber Pakhtunkhwa, Pakistan; g Department of Biochemistry, University of Malakand, Chakdara, Dir Lower, KPK, 18800, Pakistan; h Department of Pharmacognosy, College of Pharmacy, King Saud University, Riyadh, Saudi Arabia; i Department of Pharmaceutical Chemistry, College of Pharmacy, King Saud University, Riyadh, Saudi Arabia; j Department of Chemistry, University of Liverpool, UK

**Keywords:** Aurone derivatives, Analgesic, IC_50_, Molecular docking, Opioid mechanism, LOX, COX-2

## Abstract

In the current study, seven (7) aurone derivatives (ADs) were synthesized and employed to *in-vitro* LOX and COX-2 assays, *in-vivo* models of acetic acid-induced mice writhing, formalin-induced mice paw licking and tail immersion test to evaluate their analgesic potential at the doses of 10 mg and 20 mg/kg body weight. Molecular docking was performed to know the active binding site at both LOX and COX-2 as compared to standard drugs. Among the ADs, 2-(3,4-dimethoxybenzylidene)benzofuran-3(2*H*)-one (**WE-4**)possessed optimal LOX and COX-2 inhibitory strength (*IC*_*50*_=**0.30 μM** and **0.22 μM**) as compared to standard (Zileuton*IC*_*50*_ = 0.08 μM, Celecoxib*IC*_*50*_ = 0.05 μM). Similarly in various pain models compound **WE-4** showed significantly (p < 0.05) highest percent analgesic potency as compared to control at a dose of 20 mg/kg i.e**. 77.60 %** analgesic effect in acetic acid model, **49.97 %** (in Phase-1) and **70.93 %** (inPhase-2) analgesic effect in formalin pain model and **74.71 %** analgesic response in tail immersion model. By the administration of Naloxone, the tail flicking latencies were reversed (antagonized) in all treatments. The **WE-4** (at 10 mg/kg and 20 mg/kg**)** was antagonized after 90 min from **11.23** ± **0.93** and **13.41** ± **1.21** to **5.30** ± **0.48** and **4.80** ± **0.61** respectively as compared to standard Tramadol (from 17.74 ± 1.33 to 3.70 ± 0.48), showing the opiodergic receptor involvement. The molecular docking study of ADs revealed that **WE-4** had a higher affinity for LOX and COX-2 with docking scores of **-4.324** and **-5.843** respectively. As a whole, among the tested ADs, compound **WE-4** demonstrated excellent analgesic effects that may have been caused by inhibiting the LOX and COX-2 pathways.

## 1 Introduction

Pain serves as a defense mechanism against injury and helps prevent further damage to the body. However, if nociceptors, which are pain receptors, are continuously or excessively stimulated, it can lead to chronic pain, which can significantly impact an individual's quality of life [1,2]. The pain pathway is intricate and activates numerous ion channels, receptors, and neurotransmitters [3]. Inflammatory mediators, such as cytokines and chemokines, activate nociceptors, pain-sensing nerve fibers, which transmit pain signals to the spinal cord and brain [4]. Conversely, pain also activates immune cells and promotes the release of pro-inflammatory molecules, leading to the development of chronic inflammation [5].

Conventional analgesics primarily regulate pain transduction and transmission in nerve cells and are only partially effective in slowing the progression of illness [4]. Non-steroidal anti-inflammatory drugs (NSAID) are frequently used in inflammation. Both selective or non-selective COX inhibitors interact with the arachidonic acid pathway and approximately all of them have a number of undesirable, often severe major adverse effects including gastro intestinal ulcer, bleeding, heart and renal diseases [6,7]. The lipoxygenase (LOX) cascade yields leukotrienes and lipoxins, which are linked with a variety of operations such as leukocyte activation and adherence to the endothelium of vessels, bronchial asthma, edema development, and injury to the lining of the stomach [[8], [9], [10]]. Dual COX/LOX inhibitors may offer various therapeutic benefits in the context of anti-inflammatory activity, enhanced the gastric protection, and an improved cardiovascular profile when compared to conventional NSAIDs, as demonstrated by numerous research studies in the field of dual-acting COX/LOX inhibitors [11,12]. Same is the case with opioid analgesics, sometimes causing the most feared adverse effects, respiratory depression [13], sexual dysfunction [14], abuse and addiction [15] and immune system suppression [16].

Hence, the exploration of new and comparatively safe analgesic drugs to restrict these side effects can provide better alternatives to NSAIDs and opiates. Flavonoids are naturally generated secondary metabolites of polyphenolic compounds mostly from plant origin [17]. They have been linked to a wide range of health advantages and are well known for their anti-inflammatory, anti-arthritic, antiasthmatic, neuroprotective, anti-oxidant, antidiabetic, cardiotonic and anticancer qualities [[18], [19], [20]]. Many different foods, including fruits, vegetables, tea, and wine, contain flavonoids. Anthocyanins, flavones, flavonols, and flavanones are some frequent subclasses of flavonoids.

Aurones are polyphenols belonging to flavones, a subgroup of flavonoids, found in flowers, fruits and vegetables as secondary metabolites and give bright yellow colour to some fruits and flowers [21,22]. A number of different aurones are identified with different patterns of substitution which make them potentially effective for therapeutic use [21,23].

These derivatives have shown to exhibit various biological activities like antioxidants [24], as tyrosinase inhibitors [25], and inhibit kinase enzymes to stop proliferation, thus act as anticancer [26,27]. Moreover, various derivatives and analogues of aurones have been evaluated for analgesic and anti-inflammatory activities [28,29].

Aurone derivatives like sulfuretin, have been shown to possess anti-inflammatory activity. It works by inhibiting the production of prostaglandins E2 and nitric oxide [29]. Bisaminomethylated aurone is synthesized and tested against pro-inflammatory mediators i.e. tumor necroting factor alpha (TNF-α) and interleukin-6 (IL-6), and have shown excellent results against these mediators [28]. Some aurone derivatives show antibacterial and antifungal effects, causing inhibition of bacteria (mostly gram positive) and fungi while some bacterial and fungal infections lead to acute and chronic inflammation and pain, hence such infections have a correlation with inflammation associated with pain [28,30]. Thus aurone derivatives are synthesized and tested for anti-inflammatory activity [24,28,29,31,32] and inflammation has a co-relation with pain which is characterized by redness, swelling, heat and pain [33].

**Fig. 1:**
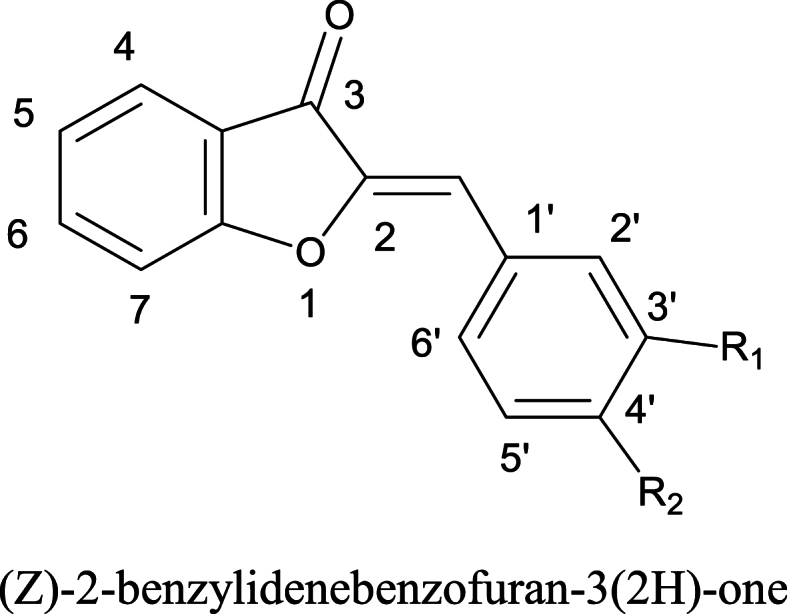
General structure of aurone.

Due to various medicinal properties, SAR studies, interact with specific biological pathways or molecular targets, chemical modification to optimize pharmacokinetic study, as a natural product and their derivatization, urgency to address unmet medical needs and emerging research trends of aurones and further more limited analgesic options, sever adverse effects associated with existing analgesics, target specific pain pathways, and limited research on aurones (as presented by generalized structure in Fig. 1) make this study possible to synthesize new aurone derivatives (ADs) and subject to molecular docking to show specific binding sites with enzymes responsible to initiate the inflammation and pain. Furthermore, to evaluate the anti-inflammatory and analgesic potential of these unreported ADs in *in-vitro;* LOX and COX-2 inhibitory assays, and *in-vivo;* acetic acid induced writhing model, formalin induced paw licking model, and to evaluate opioid analgesic mechanism by mice tail immersion model. The novelty of this study lies in its ability to contribute new and unique information or insights to the existing body of scientific knowledge.

## 2 Materials and methods

### 2.1 Chemicals

Commercially available and analytical grade chemicals were used. Carrageenan, Indomethacin, 2-hydroxyacetophenone, substituted benzaldehyde, dichloromethane (DCM), COX-2 enzyme, arachidonic acid (AA), dichlorofluorescindiacetate, silica and DMSO (Sigma Aldrich, Pakistan), Sodium chloride, calcium chloride, acetic acid and formalin (Spectrum Chemicals Pakistan), Soybean 15-LOX enzyme (Cayman Chemicals), Diclofenac Sodium, Morphine and Naloxone (Lahore Chemical and Pharmaceutical Works Pvt. Ltd, Pakistan).

### 2.2 Synthesis of aurones

**Fig. 2:**
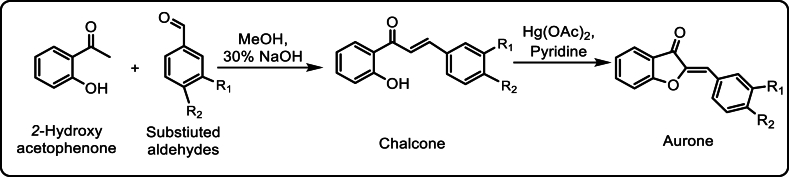
General scheme of synthesis of aurones.

Aurone derivatives were synthesized in two steps according to previously reported procedure [34] as summarized in Fig. 2. In *first step*; substituted chalcones were prepared from equimolar 2-hydroxyacetophenone and substituted benzaldehyde in menthol/ethanol by adding 20 % of 25 ml of ethanolic potassium hydroxide (KOH) and stirred this mixture at room temperature. To monitor the reaction, thin layer chromatography (TLC) was performed at different stages of the reaction to confirm the synthesis of the said chalcone. Once it was confirmed, the reaction was added with a 10 % acidic (HCl) solution to stop it. The mixture was cooled and yellow precipitates were formed, filtered and washed with 10 % HCl solution and kept it for drying in sodium sulphate (Na_2_SO_4_).

In the second step; the prepared substituted chalcones were dissolved in 10 ml dimethyl sulfoxide (DMSO) in the presence of mercuric acetate. The mixture was added in a reflux condenser and run for about 6 h. For confirmation of the reaction, TLC was performed with the same ratio and same mobile phase as stated earlier. The mixture was poured on ice water, a solid mass appeared, separated, filtered, washed with water, dried and crystallized with ethanol. Table 1 summarizes the various physicochemical characteristics of the synthesized compounds.

**Table 1:** Chemical structures and characterization data of the Synthesized Compounds.

S. No	Chemical Structure	Characterization Data	References
**WE-1**		Yield, 22.8 %; yellow crystalline; mp = 98–99 °C. ^1^H NMR (270 MHz, CDCl_3_): *δ* 7.89–7.93 (2H, m, C2’, C6’), 7.81 (1H, dd, J) 7.6, 1.3 Hz, C4), 7.65 (1H, ddd, J) 8.3, 7.3, 1.3 Hz, C6), 7.36–7.49 (3H, m, C3’, C4’, C5’), 7.32 (1H, d, J) 8.3 Hz, C7), 7.21 (1H, dd, J) 7.6, 7.3 Hz, C5), 6.89 (1H, s, benzylic).^13^C NMR (270 MHz, CDCl_3_): *δ* 184.7, 166.2, 147.0, 136.8, 132.4 (overlapped), 131.5, 129.8 (overlapped), 128.9, 124.7, 123.5, 121.7, 113.0, 112.9. DEIMS (70 eV) *m*/*z* (relative intensity): 222 (M+, 100 %), 134 (36.3 %), 120 (19.2 %). HREIMS *m*/*z* (M+), 222.0688; calcd for C_15_H_10_O_2_, 222.0681.	[35]
**WE-2**		M.P. 155–159 °C; 1H NMR (400 MHz, CDCl_3_): *δ* 7.86 (dd, *J* = 8, 2 Hz, 2H),7.83–7.80 (m, 1H), 7.68 (ddd, *J* = 8, 7, 1 Hz, 1H), 7.43 (dd, *J* = 8, 2 Hz, 2H), 7.34 (d, *J* = 8 Hz, 1H), 7.27–7.22 (m, 1H), 6.84 (s, 1H); **13C NMR** (100 MHz, CDCl_3_): *δ* 184.4, 166.3, 147.6, 138.2, 136.5, 132.6, 130.8, 129.2, 124.8, 123.7, 121.5, 113.0, 111.6; MS (ESI) *m*/*z* = 257/259.	[36]
**WE-3**		Yield, 75.3 %; yellow crystal; mp, 120–122 °C. ^1^H NMR (270 MHz, CDCl_3_): *δ* 7.88 (2H, d, J) 8.7 Hz, C2’, C6’), 7.80 (1H, dd, J) 7.6, 1.3 Hz, C4), 7.62 (1H, ddd, J) 8.3, 7.3, 1.3, Hz, C6), 7.31 (1H, dd, J) 8.3, 0.6 Hz, C7), 7.20 (1H, ddd, J) 7.6, 7.3, 0.6 Hz, C5), 6.98 (2H, d, J) 8.7 Hz, C3’, C5’), 6.88 (1H, s, benzylic), 3.86 (3H, s, Ar-OCH3).^13^C NMR (270 MHz, CDCl_3_): *δ* 184.5, 165.9, 161.1, 145.9, 136.4, 133.4 (overlapped), 125.1, 124.5, 123.2, 122.0, 114.5 (overlapped), 113.3, 112.8, 55.4. DEIMS (70 eV) *m*/*z* (relative intensity): 252 (M+, 100 %), 237 (29.4 %), 221 (38.5 %), 135 (62.8 %), 77 (73.5 %). HREIMS *m*/*z* (M+), 252.0772; calcd for C_16_H_12_O_3_, 252.0786.	[35]
**WE-4**		Yield, 65.8 %; yellow crystal; mp, 175–178 °C. ^1^H NMR (270 MHz, CDCl_3_): *δ* 7.80 (1H, dd, J) 7.6, 1.3 Hz, C4), 7.63 (1H, ddd, J) 8.3, 7.2, 1.3 Hz, C6), 7.53 (1H, d, J) 2.0 Hz, C2’), 7.49 (1H, dd, J) 8.3, 2.0 Hz, C6’), 7.30 (1H, d, J) 8.3 Hz, C7), 7.21 (1H, dd, J) 7.6, 7.3 Hz, C5), 6.94 (1H, d, J) 8.3 Hz, C5’), 6.89 (1H, s, benzylic), 3.97 (3H, s, Ar-OCH3), 3.94 (3H, s, Ar-OCH3).^13^C NMR (270 MHz, CDCl_3_): *δ* (ppm) 184.3, 165.8, 151.0, 149.2, 146.0, 136.4, 126.0, 125.4, 124.6, 123.3, 122.0, 114.1, 113.5, 112.8, 114.4, 56.0 (overlapped). DEIMS (70 eV) *m*/*z* (relative intensity): 282 (M+, 63.9 %), 267 (27.3 %), 251 (17.9 %), 134 (59.7 %), 105 (64.7 %). HREIMS *m*/*z* (M+), 282.0886; calcd for C_17_H_14_O_4_, 282.0892.	[35]
**WE-5**		Yellow solid (67 % yield); Mp 288–289 °C; ^1^H NMR (500 MHz, DMSO‑*d*_6_) *δ* 4.89 (2H, s, 6-OCH_2_COOH), 6.75–6.94 (2H, m, H-5, 2a), 7.09 (1H, s, H-7), 7.61–7.74 (1H, m, H-4), 7.87–8.10 ppm (4H, m, H-2′, 3′, 5′, 6′);^13^C NMR (125 MHz, DMSO‑*d*_6_) *δ* 65.24, 98.00, 109.59, 113.14, 114.09, 125.71, 129.52, 129.72, 131.06, 136.17, 148.12, 166.00, 166.86, 167.94, 169.40, 181.70 ppm; IR (KBr): *ν*_max_ 3542, 3064, 1749, 1691, 1605, 1447, 1277, 1104, 1056 cm^−1^; MS (APCI) *m*/*z* (%): 341.2 (100) [M+H]^+^. Anal. Calcd for C_16_H_10_O_4_: C, 63.53; H, 3.55. Found: C, 63.39; H, 3.68.	[37]
**WE-6**		Yellow crystalline solid; M.P. 126–128 °C; Yield: 68 %; R_f_ = 0.6; UV–Vis *λ*_max_ (Acetone) = 398 nm; FTIR (cm^−1^): 3092, 2832, 1706, 1642, 1591, 1413, 1292, 1045, 880; “^1^H NMR (600 MHz, DMSO‑*d*_6_): *δ* 10.23 (s, 1H, –CHO), 7.95 (d, *J* = 12.0 Hz, 2H, Ar–H), 7.79–7.76 (m, 1H, Ar–H), 7.75 (d, *J* = 12.0 Hz, 2H, Ar–H), 7.52 (d, *J* = 6.0 Hz, 1H, Ar–H), 7.35 (s, 1H, –C=C–H), 7.32 (dd, *J* = 12.0, 6.0 Hz, 1H, Ar–H), 7.25 (dd, *J* = 12.0, 6.0 Hz, 1H, Ar–H);^“13^C NMR (151 MHz, DMSO‑*d*_6_): *δ* 191.4, 183.1, 165.3, 144.9, 144.8, 137.8, 135.2, 135.0, 134.8, 133.7, 128.8, 124.6, 124.4, 122.0, 113.5, 107.4; EI-HRMS of [M]^+**·**^: Calcd. for C_16_H_10_O_3_ 250.0629; found 250.0615.	[34]
**WE-7**		Light yellow powder; M.p. 221–223 °C; Yield: 78 %; R_f_ (Cyclohexane: Ethyl acetate 3:1) = 0.52; UV–Vis l_max_ (CH_2_Cl_2_) = 429 nm; FTIR (cm^−1^): 3102, 2970, 1717, 1660, 1470, 1290, 773, 689; “^1^H NMR (600 MHz, DMSO‑*d*_6_): *δ* 8.59 (d, *J* = 6.0 Hz, 1H, Ar–H), 8.36 (d, *J* = 6.0 Hz, 1H, Ar–H), 8.21 (dd, *J* = 12.0, 6.0 Hz, 2H, Ar–H), 7.47 (dd, *J* = 12.0, 6.0 Hz, 2H, Ar–H), 7.26 (s, 1H, –C=C–H);^13^C NMR (151 MHz, DMSO‑*d*_6_): *δ* 181.0, 164.7, 163.0, 156.6, 145.8, 143.8, 134.8131.6, 128.4, 128.3, 128.2, 126.7, 117.0, 116.9, 114.7; accurate mass (EI-MS) of [M]^+**·**^: Calcd. for C_15_H_7_^35^ClFNO_4_ 319.0047; found 319.0040.	[38]

### 2.3 Invitro enzyme inhibition

Aurone derivatives were assessed for their potential to inhibit the cyclooxygenase 2 (COX-2) and lipoxygenase enzymes (LOX) [39].

#### 2.3.1 COX-2 inhibitory assay

COX-2 inhibition was measured spectrophotometrically at 550 nm after the formation of oxidized form of tetramethlenephenylenediamine (TMPD). This assay was conducted according to previously reported procedure [39]. Seven (7) aurone derivatives (each 17.45 μM, 33.3 μM, 49.6 μM) were tested against COX-2 enzyme in 96 well plate. The reaction contained 10 μL COX-2 enzymes (100 μM in diluted in DMSO), 10 μL haem of 2.2 mM diluted in DMSO, tris buffers (pH. 8, 100 μl) and drug samples according to the above stated procedure. The reaction was started by the addition of freshly prepared TMPD and methanolic arachidonic acid (AA). Celecoxib is used as a reference drug in this assay. After 5 min of incubation at 25–28C, the absorbance was measured at 550 nm and inhibition was calculated.

#### 2.3.2 LOX inhibitory assay

The assay was performed according to previously reported procedure [39]. All test compounds (WE-1 to WE-7) were tested against 5-LOX at 100 μM and compared to standard (Zileutone). Each well of the microplate was incorporated with buffer, 5-LOX enzyme, arachadonic acid, dichlorofluorescin diacetate (DCFDA), test compounds and adenosine triphosphate (ATP) in specified concentration described by Md Idris et al., 2022. Enzyme 5-LOX and DCFDA were incubated for 5 min. After the addition of the test compound, it was further incubated for 10 min. By the addition of substrate arachadonic acid and ATP, the reaction was initiated. By replacing the test compounds with DMSO, 5-LOX activity was measured by microplate reader (Polar Star Omega, Germany) spectroscopically at a wavelength of 485 and 520 nm and inhibition was calculated.

### 2.4 Animals and ethical approval

Male Swiss albino BALB/c mice weighing 20±2 g were obtained from the Veterinary Research Institute (VRI) in Peshawar, Khyber Pakhtunkhwa (KP), Pakistan. They were housed in standard experimental conditions within the experimental animal house at the Department of Pharmacy, Abdul Wali Khan University in Mardan, KP, Pakistan. The mice were provided with standard feed and water *ad libitum* and subjected to a 12-h light-dark cycle. Prior to the experiment, they were fasted for 6 h. Fresh animals were used for each experiment. Ethical approval was approved from University Ethical Committee and Dean Faculty of Life Sciences, Abdul Wali Khan University, Mardan under ethical approval No. EC/AWKUM/2022/04/26. All ethical guidelines established for laboratory animals in 1979 were strictly followed during the study [40].

### 2.5 Acute toxicity

In order to evaluate any potential toxicity of the aurone derivatives as described by Shoaib et al., 2016 [41], an acute toxicity test was conducted. Male Albino mice (n = 8) were administered different doses (100, 500, and 1000 mg/kg, p. o.) of the compounds, while the control group received saline (10 ml/kg). The groups were carefully observed for 24 h for any visible effects (mortality).

### 2.6 Analgesic activity

The analgesic activity of the aurone derivatives was evaluated through several in-vivo studies to determine their potential as pain-relieving agents. These studies were conducted to assess the effectiveness of the derivatives in alleviating pain. The results aimed to provide insight into the analgesic properties of these compounds.

#### 2.6.1 Mice grouping and dosing

The animals were divided into various groups I-XVI consisting of 8 experimental animals for control, sample compounds **(WE-1** to **WE-7**) and standard. Group I: Control, Group II-XV: **WE-1** to **WE-7** (sample compounds) at dose of 10 mg/kg and 20 mg/kg and Group XVI: Standard. The control (Group I) was given normal saline. Group II-XV received 10 mg/kg and 20 mg/kg of test sample (**WE-1** to **WE-7**). Standard (Group XVI) received Diclofenac Sodium 10 mg/kg in case of acetic acid induced writhing test, Indomethacin 10 mg/kg for formalin induced paw licking test, Morphine 2 mg/kg and Tramadol 10 mg/kg for tail immersion test.

#### 2.6.2 Writhing test

The writhing test, following a previously established method [42], was conducted to assess the analgesic potential of the aurone derivatives. The mice were orally administered samples compounds (WE-1 to WE-7)and standard (Diclofenac sodium 10 mg/kg) 1 h before intraperitoneal administration 10 ml/kg of 1 % acetic acid. Five minutes after the acetic acid administration, the numbers of writhing episodes were counted for 10 min, and the percentage of inhibition was subsequently calculated.

#### 2.6.3 Formalin induced method

The acute peripheral and centrally mediated analgesic activity of aurone derivatives was evaluated using the formalin-induced pain method, as described [42]. Samples compounds (**WE-1 to WE-7**) and a standard (Indomethacin 10 mg) were orally administered 30 min prior to 20 μl of 1 % formalin injection (prepared in 0.9 % saline) in right hind paw of the mice and immediately placed in a clear box to observe the animal's reaction. Paw licking or biting within 0–5 min indicated Phase 1, while animal reactions observed between 15 and 30 min indicated Phase 2. The number of paw licking or biting events was compared to the control group, and the percentage of inhibition was calculated.

#### 2.6.4 Tail immersion test

The experiment was conducted with a little modification, following the methodology described in the research conducted by Shoaib et al., 2017 [42]. Sample compounds (**WE-1 and WE-7**) and standard drugs (Morphine 2 mg/kg and Tramadol 10 mg/kg), were given orally to the respective groups of mice. The anti-nociceptive activity was assessed 60 min after the administration of the sample's compounds and 30 min after Morphine and Tramadol administration. The mice's tail tips were immersed up to 2 cm in a water bath maintained at a thermostatically controlled temperature of 55 °C ± 1 °C. The cut-off time was set at 25 s, and the time taken for the mice to flick their tails out of the water was recorded at 30, 60, 90, and 120-min intervals. An increase in the time of tail immersion was compared to the control group to determine the analgesic effect of the tested drugs. The percentage of inhibition was calculated at the time interval showing the highest effect.

### 2.7 Assessment of opioid mechanism

To verify the involvement of the opioid mechanism, following the approach outlined in the published study [43]. Four aurone derivatives (**WE-2, WE-3, WE-4, and WE-7**) were chosen among the sample compounds based on their analgesic potential in this study. These derivatives were assessed for their potential opioid activity within the control, sample compounds and standard groups using the tail immersion test. Naloxone, an opioid antagonist, was administered at a dose of 2 mg/kg b. w along with all sample compounds (**WE-1 to WE-7**) and the standard drug (Tramadol).

### 2.8 Kinetic assay

The type of enzyme inhibition by compound (**WE-4**) was determined using the continuous spectrophotometric rate determination method. Various concentrations of the substrate (AA) were prepared in a Tween-20 solution within a 200 mM borate buffer at pH 9.0. A fixed concentration of LOX enzyme (10,000 units/ml) was prepared in a separate 200 mM borate buffer at pH 9.0. The enzyme kinetics were analyzed by employing the Lineweaver and Burk method [44] while examining the substrate at different concentrations in the presence or absence of compound (**WE-4**).

### 2.9 Molecular docking

Docking studies of all the synthesized compounds were performed to investigate the potential to be dual inhibitors and possible binding mode in the binding sites of COX-2 and human LOX enzymes using Maestro Schrödinger (V 2017–2, Schrödinger, LLC, Oregon, USA). The X-ray crystal structures of human COX-2, and human LOX with PDB IDs of 5F19, and 3O8Y respectively, were retrieved from the Protein Database Bank (PDB) (https://www.rcsb.org/pdb/home/home.do) [45]. The structures of all the synthesized ligands were prepared by the LigPrep module in Schrödinger in their neutral form and optimized by the OPLS3e force field. Based on the highest resolution (2.04 Å) the PDB ID 5F19 represented COX-2 in this study and was prepared by method with little modifications as reported by Md Idris et al. [39]. The structures of all proteins were prepared using the Protein Preparation Wizard in Maestro by removing water molecules beyond 5 Å from het atoms and adding hydrogens to set protonation states suitable for pH 7. The receptor grid box was defined with the active site water molecule to explore potential interactions between the ligand and water-mediated interactions in the active site. Water molecules can play a crucial role in mediating protein-ligand interactions, as evidenced by their ability to form multiple hydrogen bonds and also aid in stabilization of protein-ligand complexes [46,47]. The Glide module of Maestro Schrodinger suite performed docking study, using an extra-precision (XP) method with default parameters and reported with top 15 highest ranked docked poses for each ligand. Ligand binding interaction was used to generate 2D, 3D images and visual inspection was carried out for docked complexes of all synthesized ligands.

### 2.10 Statistical analysis

All the results obtained were expressed as mean ± SEM (standard error mean) of eight animals (n = 8), percent inhibition was also carried out for results where it was necessary. Statistical analysis in the form ANOVA was implemented followed by post hoc Dunnet's test for multiple comparisons between and among groups. Effect of results were considered to be significant at level of P < 0.05.

### 2.11 ADMET assay

ADMET properties are determined using MolSoft tool as previously reported. The Lipinski Rule of Five is required to guarantee drug-like qualities to evaluate the oral acceptability of drugs and provides a framework for calculating the pharmacokinetics and bioavailability of given molecules on the basis of various sets of physicochemical standards [48].

## 3 Results

### 3.1 In-vitro anti-inflammatory studies (LOX and COX-2)

**Fig. 3:**
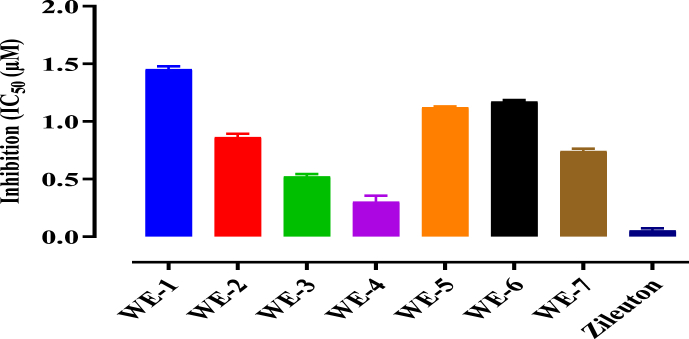
Test compounds (WE-1 toWE-7) Showing inhibition of 15 LOX.

**Fig. 4:**
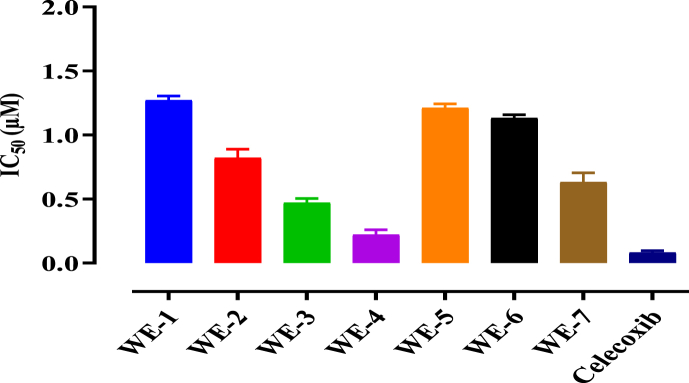
Test compounds WE (1–7) Showing inhibition (IC_50_) of COX-2.

The results of the investigation into the inhibitory effects of compounds **WE1** to **WE-7** on LOX and COX-2 enzymes are presented in Table 2 whereas graphically in Fig. 3 (for LOX), and Fig. 4 (for COX-2). Among the tested compounds, WE-4 exhibited the most significant inhibitory effect, with an IC_50_ value of 0.3 μM for LOX and 0.22 μM for COX-2. The standard drugs, Zileuton and Celecoxib, were used for LOX and COX-2, respectively, with IC_50_ values of 0.05 μM and 0.08 μM. Additionally, other test compounds, namely WE-3, WE-7, and WE-2, also demonstrated significant inhibition of both LOX and COX-2, as indicated by their respective IC_50_ values presented in Table 2.

**Table 2:** IC_50_ Values of test compounds (**WE1** to **W7**).

COMPOUND	LOX (IC_50_ μM)	COX-2 (IC_50_ μM)
WE-1	1.45	1.27
WE-2	0.86	0.82
WE-3	0.52	0.47
WE-4	0.30	0.22
WE-5	1.12	1.21
WE-6	1.17	1.13
WE-7	0.74	0.63
Zileuton	0.05	–
Celecoxib	–	0.08

### 3.2 Acute toxicity test

The aurone derivatives **WE-1 to WE-7** were determined to be safe at doses of 100, 500, and 1000 mg/kg body weight (b.w). All the animals remained in a normal state and exhibited no signs of toxicity throughout the 24-h assessment period.

### 3.3 Acetic acid induced writhing in mice model

**Fig. 5:**
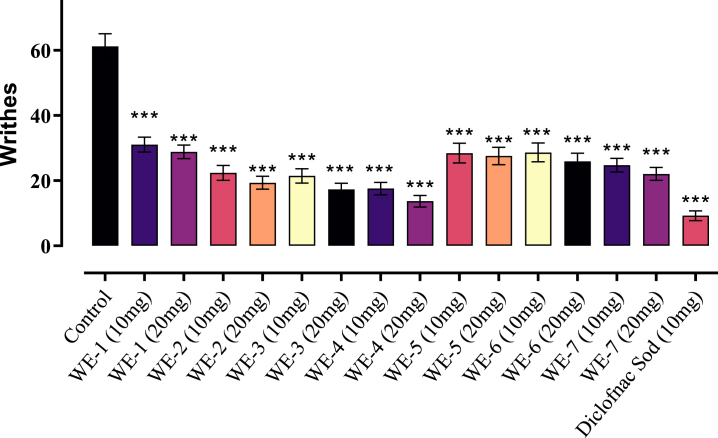
Acetic-acid induced analgesic activity of (**WE-1 to WE-7**). Mean ± SEM, n = 8. ***p < 0.001 vs. the control group. Dunnett's multiple comparison tests was applied after a one-way ANOVA.

In this model, seven compounds (**WE-1 to WE-7**) were tested at doses of 10 and 20 mg/kg b. w, along with control and standard groups, as depicted in Fig. 4. The observed effect was statistically significant (p < 0.05). Among the compounds, **WE-4** exhibited the highest effect, with values of 13.71 ± 1.77 (77.60 %) and 17.57 ± 1.94 (71.30 %) at doses of 20 mg/kg and 10 mg/kg, respectively, in mice, compared to the control value of 61.22 ± 3.90. Additionally, other compounds, such as **WE-2** and **WE-3**, demonstrated analgesic effects of 63.44 %, 64.94 %, and 68.37 %, 71.72 % at doses of 10 and 20 mg/kg body weight respectively. Moreover, at a dose of 20 mg/kg, drug **WE-7** also exhibited more than 60 % inhibition of writhing (pain) in mice. As a standard drug, diclofenac sodium displayed an effect of 9.24 ± 1.51 (84.90 %) at a dose of 10 mg/kg b. w (Fig. 5).

### 3.4 Formalin induced paw licking response

**Fig. 6:**
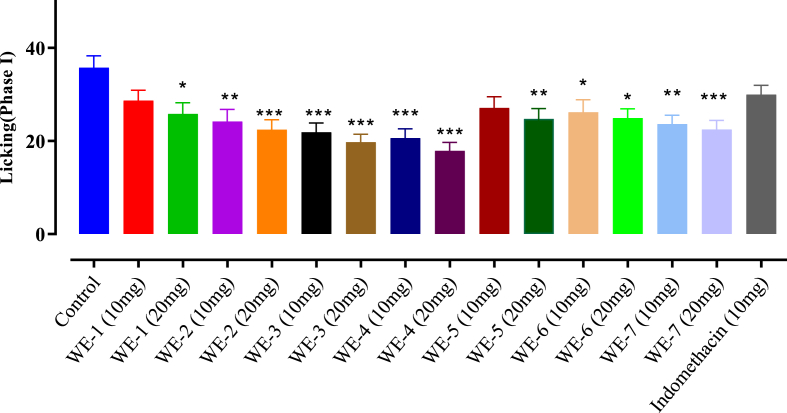
Formalin induced paw licking **Phase-I** analgesic activity of **WE** (**1**–**7**). Mean ± SEM, n = 7. *p < 0.05, **p < 0.01 and ***p < 0.001 vs. the control group. Dunnett's multiple comparison tests was applied after a one-way ANOVA.

**Fig. 7:**
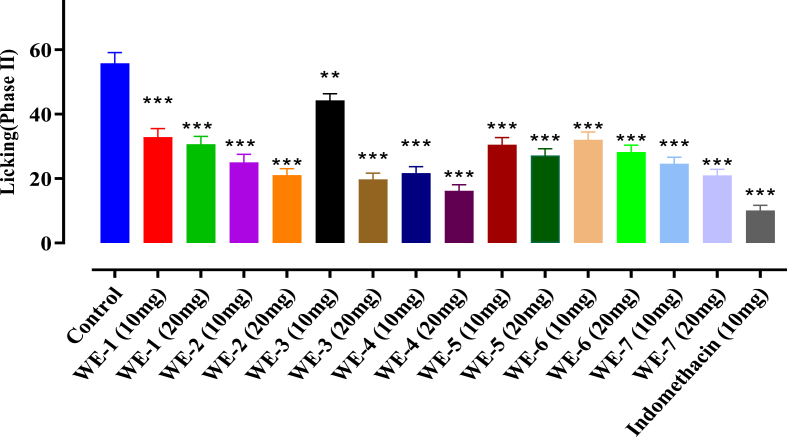
Formalin induced paw licking **Phase-II** analgesic activity of **WE** (**1**–**7**). Mean ± SEM, n = 8. **p < 0.01 and ***p < 0.001 vs. the control group. Dunnett's multiple comparison tests was applied after a one-way ANOVA.

The results presented in Fig. 6, Fig. 7, demonstrated that at a dose of 20 mg/kg b. w, **WE-4** significantly (p < 0.05) reduced the number of formalin-induced paw licking in mice during the late phase (Phase II), with a value of 16.22 ± 1.86 (70.96 %) compared to the control value of 55.81 ± 3.29. Furthermore, **WE-4** exhibited the highest percentage inhibition (49.97 %) among the tested compounds during the initial phase (Phase I). Other compounds, namely **WE-2**, **WE-3**, and **WE-7**, also produced over 60 % inhibition of paw licking at a dose of 20 mg/kg b. w during the late phase. As the standard drug, indomethacin at a dose of 10 mg/kg showed the highest percentage inhibition (81.85 %) in Phase II.

### 3.5 Tail immersion test

**Fig. 8:**
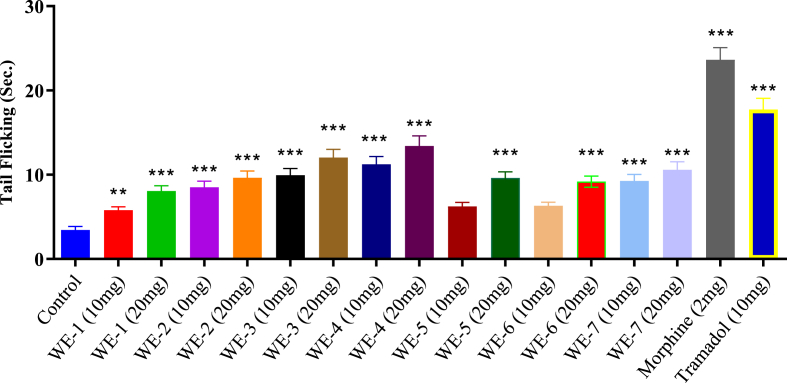
Heat induced **Tail Flicking Latency** (analgesic activity) of **WE** (**1**–**7**). Mean ± SEM, n = 8. **p < 0.01 and ***p < 0.001 vs. the control group. Dunnett's multiple comparison tests was applied after a one-way ANOVA.

In Fig. 8, the results indicated that all seven test compounds (**WE-1** to **WE-7**), along with control and standard groups, were examined. Among these, WE-4 (at a dose of 20 mg/kg) exhibited the most significant (p < 0.05) outcome, with the highest percentage inhibition of pain (74.31 %) and an increased latency of 13.41 ± 1.21 after 90 min, compared to the control. Another compound, WE-3, also demonstrated notable activity with 71.36 % at a dose of 20 mg/kg b. w. Morphine and Tramadol were employed as standard drugs and displayed the highest percentage inhibitions of 85.42 % and 80.57 %, respectively.

### 3.6 Assessment of opioid mechanism

Upon administration of Naloxone, the tail flicking latencies observed in all drugs were reversed, indicating antagonistic effects. Specifically, in the case of **WE-4**, at doses of 10 mg/kg and 20 mg/kg, the latencies decreased from 11.23 ± 0.93 and 13.41 ± 1.21 to 5.30 ± 0.48 and 4.80 ± 0.61, respectively, after 90 min. This effect was comparable to the standard drug Tramadol, which reduced the latency from 17.74 ± 1.33 to 3.70 ± 0.48, as shown in Table 3.

**Table 3:** Assessment of opioid mechanism via tail immersion test.

Treatment dose		Latency response			
		30 min	60 min	90 min	120 min
Control		3.14 ± 0.31	3.17 ± 0.41	3.31 ± 0.44	3.20 ± 0.29
**WE-2** + **Naloxone**	10 + 2	3.61 ± 0.41	4.11 ± 0.40	4.03 ± 0.41	3.19 ± 0.32
	20 + 2	3.70 ± 0.39	4.30 ± 0.51	4.59 ± 0.60	3.80 ± 0.55
**WE-3** + **Naloxone**	10 + 2	3.66 ± 0.41	4.60 ± 0.49	4.71 ± 0.66	4.01 ± 0.61
	20 + 2	3.35 ± 0.49	4.76 ± 0.51	5.14 ± 0.61	4.15 ± 0.56
**WE-4** + **Naloxone**	10 + 2	3.60 ± 0.61	4.49 ± 0.43	5.30 ± 0.48	3.80 ± 0.49
	20 + 2	3.41 ± 0.48	4.67 ± 0.60	4.80 ± 0.61	4.03 ± 0.57
**WE-7** + **Naloxone**	10 + 2	3.48 ± 0.46	4.11 ± 0.48	4.11 ± 0.40	3.40 ± 0.60
	20 + 2	3.50 ± 0.67	4.35 ± 0.61	5.01 ± 0.60	3.51 ± 0.66
**Tramadol** + **Naloxone**	10 + 2	3.53 ± 0.44	4.60 ± 0.59	3.70 ± 0.48	3.48 ± 0.44

### 3.7 Enzyme kinetic study

**Fig. 9:**
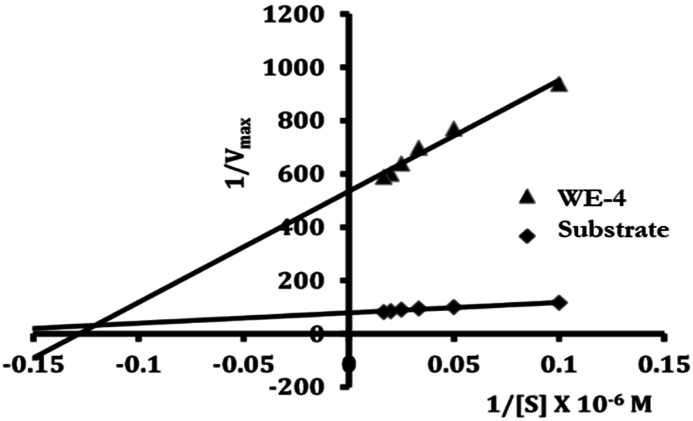
*In vitro* LOX enzyme kinetics demonstrating non-competitive inhibition by compound **WE-4**.

The Lineweaver-Burk plot was utilized to ascertain the nature of enzyme inhibition in the study. To investigate the substrate-dependent enzyme kinetics of the active compound (**WE-4**), the researchers examined its effects on the enzyme activity (as depicted in Fig. 9). By comparing the obtained 1/Vmax (y-intercept) values in the presence and absence of the inhibitor, they were able to determine different values, and subsequently calculated the inhibition constant (K_i_). The K_i_ value of WE-4 is 0.73. Analysis of the Lineweaver-Burk plot revealed that compound (**WE-4**) acts as a non-competitive inhibitor.

### 3.8 Molecular docking study

**Fig. 10:**
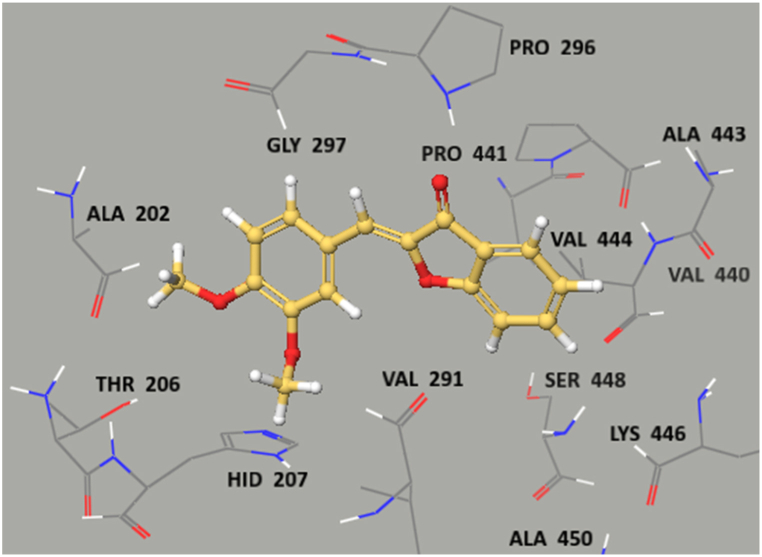
Ligand protein interactions diagram for WE-4 in catalytic pocket of human cyclooxygenase COX-2 enzyme (PDB ID 5F19).

**Fig. 11:**
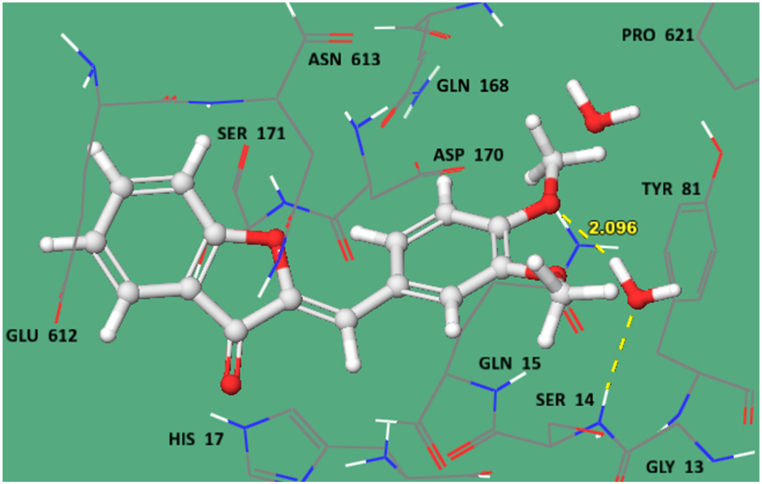
Ligand protein interactions diagram for **WE-4** in catalytic pocket of lipoxygenase human LOX enzyme (PDB ID 3O8Y).

The preferred binding interactions/poses of ligand **WE-4** in the catalytic pocket of COX-2 and human LOX were further analyzed (Fig. 10, Fig. 11). The binding energies of synthesized ligands indicated moderate to decent fittings in the active sites of target proteins (Table 4). For the COX-enzymes, the crucial amino acids involved were Ala202, Thr206, Hid 207, Val291, Pro441, Ala443, Val444, Lys446, Ser448 and Ala450 whereas for human LOX Gly 13, Ser 14,171, Gln 15,168, His 17, Tyr81, Asp 170, Glu 612, Asn 613 and Pro 621 residues are critical amino acids in forming ligand-protein complex. The active sites selected using Pfam. The **WE-4** (docking score −5.843, Table 4) protruded near the opening site of COX-2 without any interaction due to its rigid structure.

**Table 4:** Docking score of synthesized aurones **WE** (**1–7)**.

Sample codes	Human LOX (IC_50_ μM)	Docking score PDB ID 5F19	COX-2 (IC_50_ μM)	Docking score PDB ID 3O8Y	Interacting residues
**WE-1**	1.45	−4.261	1.27	−5.760	
**WE-2**	0.86	−3.211	0.82	−6.357	
**WE-3**	0.52	−4.053	0.47	−3.918	
**WE-4**	0.30	−4.324	0.22	−5.843	For 3O8Y HOH881 (2.096 Å), For 5F19 (Nill)
**WE-5**	1.12	−3.466	1.21	−4.644	
**WE-6**	1.17	−3.998	1.13	−4.568	
**WE-7**	0.74	−3.160	0.63	−6.284	
**Celecoxib**	–	–	0.041	−5.660	
**Zileuton**	0.49	−4.214	–	–	

In case of human LOX, **WE-4** (docking score −4.324) picked up a hydrogen bond (2.096 Å) with the active site water molecule of human LOX and stabilized it by surrounded hydrophobic and polar interactions (Fig. 11).

### 3.9 ADMET studies

The therapeutical effectiveness of target compounds **WE (1–7)** were evaluated using Lipinski's rule (Table 5). For an orally taken drug to absorb in body should have a molecular weight ≤500 g/mol, a donor hydrogen bond (HBD) ≤ 5, an acceptor hydrogen bond (HBA) ≤ 10, a topological polar surface area (TPSA) ≤ 140 A^2^, and molLogP value ≤ 5. All the compounds had very good drug likeness properties in accordance with Lipinski's rule. All ompounds had molLogP values below 5, indicating that these analogues have good solubility and higher membrane permeability. The results indicate that several analogues have strong drug-like characteristics and are RO5 compliant, suggesting that they have the potential to be developed and employed as drugs.

**Table 5:** ADMET studies of synthesized aurones **WE** (**1–7)**.

Compound	Molecular Formula	M.Wt ≤ 500	HBA ≤10	HBD ≤5	LogP ≤5	LogS (mol/L) ≤ - 4	TPSA ≤140 (A^2^)	Volume (A^3^)	Drug likeness Score >0	Lipinski's rule of 5 (RO5)
**WE-1**	C_15_H_10_ O_2_	222.07	2	0	3.52	−4.04	21.36	238.95	−0.42	Yes
**WE-2**	C_15_H_9_ClO_2_	256.03	2	0	4.11	−4.79	21.36	256.14	0.07	Yes
**WE-3**	C_16_H_12_O_3_	252.08	3	0	3.47	−3.82	28.91	270.79	−0.06	Yes
**WE-4**	C_17_H_14_O_4_	282.09	4	0	3.11	−3.36	36.62	302.22	−0.18	Yes
**WE-5**	C_16_H_10_O_4_	266.06	4	1	3.32	−3.48	49.77	272.05	0.20	Yes
**WE-6**	C_16_H_10_O_3_	250.06	3	0	3.0	−3.50	35.52	267.13	−0.57	Yes
**WE-7**	C_15_H_9_FO_2_	240.06	2	0	3.58	−4.16	21.36	244.86	0.01	Yes

## 4 Discussion

This study focuses on the synthesis of seven aurone derivatives, utilizing environmentally friendly chemical reactions. The synthesized derivatives were further subjected to molecular docking to identify the optimal binding site with LOX and COX-2 enzymes, thereby confirming their inhibitory potential against these enzymes. These derivatives were then evaluated for their analgesic effects in various models, demonstrating enhanced activity in both peripheral and central analgesia, encompassing both opioid and non-opioid mechanisms. To verify the involvement of the opioid mechanism, Naloxone, an opioid antagonist, was employed. The administration of Naloxone reversed the analgesic effects, as indicated by decreased tail flicking latencies, in all test drugs (WE-1 to WE-7) as well as the standard drug (Tramadol), thereby confirming the participation of opioid receptors.

The enzyme LOX, also known as lipoxygenase, catalyzes the oxidation of polyunsaturated fatty acids (PUFAs) to generate a range of reactive lipid mediators [40].

Leukotrienes, lipoxins, and hepoxilins are among the pro-inflammatory lipid mediators synthesized by LOX. These mediators play crucial roles in initiating and propagating inflammation. The expression of LOX is upregulated during inflammation, and numerous animal models have shown that inhibiting LOX can effectively reduce inflammation [49]. LOX expression has been found to be elevated in a variety of cancers, including breast, prostate, lung, and colon cancer [50]. LOX-produced leukotriene B4 (LTB4) was researched and found to induce atherosclerosis in cardiovascular disease [51]. In short, by inhibiting LOX enzyme, pro-inflammatory mediators will not be released and thus chronic inflammation will be stopped. Several research have been conducted to investigate the interaction of lipoxygenase enzymes and inhibitory ligands. Charlier (2006) provided a model of human 5-LOX interaction that highlighted the relevance of hydrophobic groups, aromatic rings, and hydrogen bond acceptors [52]. Gupta (1990) published a research on lipoxygenase inhibitors, showing the importance of hydrophobicity and substituent size in determining inhibitory efficacy [53]. Molecular docking studies reveal that inhibitory ligands interact with lipoxygenase enzymes through key binding sites, forming stable complexes. These interactions often involve hydrogen bonding, hydrophobic interactions, polarity and electrostatic forces, influencing the enzyme's active site conformation and inhibiting its catalytic function. Understanding these molecular interactions aids in designing potential therapeutic agents targeting lipoxygenase activity.

As a result of the LOX inhibitory experiment, it was discovered that aurone derivatives have the capacity to inhibit LOX. It means these derivatives bind with active site at LOX enzyme and block to produce inflammatory mediators which were confirmed by molecular docking; In the case of human LOX, the ligand WE-4, with a docking score of −4.324, formed a hydrogen bond (2.096 Å) with a water molecule present in the active site of human LOX. This interaction was supported by surrounding hydrophobic and polar interactions, which helped stabilize the binding of WE-4 in the active site of the enzyme.

COX-2 catalyzes conversion of arachidonic acid into prostaglandins, which are involved sometimes in the pathogenesis of inflammatory disorders such as osteoarthritis, gout, and acute pain [54]. COX-2 has also been linked to cancer growth and progression. It is overexpressed in several types of tumors, including colorectal, lung, breast, and prostatic cancers, and is linked to enhanced vascular growth, cell proliferation, and apoptosis resistance [55]. It is hypothesized and could be involved in neurological inflammation, oxidative stress, and damage to neurons in people.

In the COX-2 inhibitory assay, the aurone derivatives demonstrated the ability to inhibit this enzyme, which is a key contributor to chronic inflammation. These derivatives bind to the active site of COX-2. This binding induces conformational changes that prevent COX-2 from releasing inflammatory mediators that contribute to chronic inflammation. As a result, the aurone derivatives have the potential to mitigate the effects of chronic inflammation. This statement was supporting by molecular docking; for the COX enzymes, certain essential amino acids, including Ala202, Thr206, His 207, Val291, Pro441, Ala443, Val444, Lys446, Ser448, and Ala450, played crucial roles in the binding process. Among the ligands, WE-4 (with a docking score of −5.843 kcal/mol) positioned itself close to the entrance site of COX-2.

The acetic acid-induced writhing test in mice is a commonly used and recommended method to assess the peripheral analgesic effects of a drug. This procedure involves observing the writhing or abdominal contractions that occur due to pain in the abdominal organs of mice. The pain is caused by the production of specific prostaglandins, namely prostaglandin E2 (PGE-2) and prostaglandin F2 alpha (PGF-2α), which are derived from arachidonic acid through the action of cyclooxygenase on phospholipid tissues in the visceral region. The presence of these prostaglandins leads to inflammation, pain, and hyperalgesia, which subsequently result in the observed writhing, characterized by abdominal muscle contractions and elongation in mice [42,56,57]. The drugs or substances which decrease or stop these writhing (due to pain) in mice model are described as analgesics.

In this context, the use of aurone derivatives significantly decreases the numbers of writhing induced by acetic acid in mice, showing an analgesic effect. This means these substances are pharmacologically active which inhibits the production of prostaglandin and other pain inducing mediators (arachidonic acid, histamine, serotonin, substance P) as a whole or some of them. Hence relieved the pain and significantly decreased the numbers of writhing in mice.

The formalin-induced pain model in mice is a widely used and reliable method to evaluate the analgesic effects of drugs or substances that act centrally, peripherally, or both. In this model, the pain experienced during the initial phase (centrally) reflects the activation of pain receptors, typically lasting between 5 and 15 min. On the other hand, pain occurring in peripheral tissues during the late phase (more than 15 min) is attributed to the release of prostaglandins from arachidonic acid through the action of lipoxygenase enzymes on phospholipid tissues. Previous studies have indicated that bradykinins and substance P are released during the initial phase, while prostaglandins, serotonin, histamine, and nitric oxide are involved in the late phase of the formalin-induced pain model [42,58].

The aurone derivatives were evaluated in this model, demonstrating a noteworthy analgesic effect on both centrally mediated pain and peripherally mediated pain. This suggests that the compounds have the capability to suppress the release of pain mediators during both phases of pain. These mediators, responsible for initiating pain during the initial phase (central) and perpetuating pain during the late phase (peripheral), are inhibited or their release is reduced by these compounds, thereby alleviating pain in the animal model.

To further confirm the centrally acting analgesic effect of the aurone derivatives, a tail immersion test was performed in a rat model. The results clearly indicated a substantial increase in the latency of tail flicking in response to the derivatives compared to the control group and the standard drug (Morphine). In order to verify the involvement of the opioid mechanism, an opioid antagonist (Naloxone) was utilized. This confirmed the blockade of opioid receptors, resulting in a decrease in tail flicking latency.

When pre-treated with naloxone, the analgesic effects of morphine and tramadol, which are centrally acting opioid analgesics, were significantly diminished. This indicates the involvement of the opioid system in the analgesic response. However, when the aurone derivatives were pre-treated with naloxone, there was reduction in the analgesic response in mice. This suggests the role of opioid receptors in the analgesic effects of the aurone derivatives.

## 5 Conclusions

In this study, seven aurone derivatives (**WE-1 to WE-7**) were synthesized and their analgesic potential was evaluated using in vitro and in vivo models. Additionally, their binding affinity to specific target sites in enzymes was analyzed through molecular docking. Remarkably, in all of the tested models, the aurone derivatives exhibited significant analgesic effects, effectively reducing the pain threshold. These findings strongly suggested the involvement of opioid receptors in the mechanism of action of these derivatives. As a result, these synthetic aurone derivatives hold promise as potential candidates deserving further investigation for the development of pain treatments. However, it is important to note that these findings represent preliminary results, and additional research is needed to fully elucidate the precise mechanisms underlying their pain-relieving properties.

Further research should focus on uncovering the specific molecular pathways and signaling mechanisms through which these aurone derivatives exert their analgesic effects. This will contribute to a more comprehensive understanding of their potential therapeutic applications in the field of pain management. Continued investigation into these synthetic compounds will aid in optimizing their efficacy, safety, and overall utility as potential treatment options for various pain-related conditions.

## Data availability statement

No data was used for the research described in the article.

## CRediT authorship contribution statement

**Muhammad Ikram:** Formal analysis, Conceptualization. **Ismail Shah:** Methodology, Formal analysis, Data curation. **Haya Hussain:** Methodology, Formal analysis, Data curation. **Ehsan Ullah Mughal:** Software, Project administration, Formal analysis, Data curation. **Nafeesa Naeem:** Funding acquisition, Formal analysis, Data curation. **Amina Sadiq:** Methodology, Investigation, Formal analysis, Data curation. **Yasir Nazir:** Methodology, Investigation, Funding acquisition, Formal analysis, Data curation. **Syed Wadood Ali Shah:** Project administration, Methodology, Conceptualization. **Muhammad Zahoor:** Writing – review & editing, Project administration, Conceptualization. **Riaz Ullah:** Resources, Project administration, Funding acquisition, Data curation, Conceptualization. **Essam A. Ali:** Investigation, Funding acquisition, Formal analysis, Data curation, Conceptualization. **Muhammad Naveed Umar:** Funding acquisition, Formal analysis, Data curation, Conceptualization.

## Declaration of competing interest

The authors declare that they have no known competing financial interests or personal relationships that could have appeared to influence the work reported in this paper.
